# Sulfur dioxide reduces hippocampal cell death and improves learning and memory deficits in a rat model of transient global ischemia/reperfusion

**DOI:** 10.22038/IJBMS.2018.29404.7106

**Published:** 2018-10

**Authors:** Fatemeh Zare Mehrjerdi, Ali Shoshtari, Fahimeh Mohseni, Hossein Khastar, Pirasteh Norozi, Yasin Asadi, Masoumeh Dadkhah, Mehdi Khaksari

**Affiliations:** 1 Neurobiomedical Research Center, Shahid Sadoughi University of Medical Sciences, Yazd, Iran; 2 Student Research Committee, School of Medicine, Shahroud University of Medical Sciences, Shahroud, Iran; 3 School of Medicine, Shahroud University of Medical Sciences, Shahroud, Iran; 4 Department of Neuroscience, Faculty of Advanced Technologies in Medicine, Iran University of Medical Sciences, Tehran, Iran; 5 Laboratory of Learning and Memory, Physiology Research Center and Physiology Department, School of Medicine, Semnan University of Medical Sciences, Semnan, Iran; 6 Addiction Research Center, Shahroud University of Medical Sciences, Shahroud, Iran

**Keywords:** Antioxidant activity, Apoptosis, Brain ischemia, Memory, Sulfur dioxide

## Abstract

**Objective(s):**

According to recent the findings, sulfur dioxide (SO_2_) is produced by the cardiovascular system, influencing some major biological processes. Based on previous research, SO_2_ exhibits antioxidant effects and inhibits apoptosis following cardiac ischemia/reperfusion. Therefore, the objective of the current study was to examine the neuroprotective impact of SO2 following global cerebral ischemia/reperfusion (I/R).

**Materials and Methods:**

Forty-eight male Wistar rats that weighed 260–300 g, were randomly allocated into 4 groups: sham group (n=12), I/R group (n=12), and I/R+SO_2_ groups (NaHSO_3 _and Na_2_SO_3_; 1:3 ratio; 5 and 10 µg/kg, respectively; for 3 days, n=12). Cerebral ischemia model was prepared by occlusion of both common carotid arteries for 20 min. Saline as a vehicle and SO_2_ donor at doses 5 µg/kg (intraperitoneally) were injected for 3 days after reperfusion. Four days after ischemia, the passive avoidance memory test was carried out in four groups, and after behavioral assessment, necrosis, apoptosis, and antioxidant enzyme analysis were carried out.

**Results:**

O_2_ treatment could significantly improve memory impairments in rats with cerebral ischemia/reperfusion (I/R) (*P*<0.05). An increase in both superoxide dismutase and glutathione and a reduction in malondialdehyde were reported in the SO_2_ group versus the ischemic group (*P*<0.05). Moreover, SO_2_ could significantly decrease necrotic and apoptotic cells in the CA1 region (*P*<0.01).

**Conclusion:**

According to the findings, SO_2_ exerts significant neuroprotective effects on cerebral I/R due to its antioxidant activity.

## Introduction

Sulfur dioxide (SO_2_) was known as an atmospheric pollutant in the past, and its toxicological effects were studied extensively (1). However, recent findings indicate that SO_2_ is produced by the cardiovascular system and affects major biological processes. Endogenous SO_2_ and its derivatives have vasorelaxant activities (2, 3). A recent study has revealed that pulmonary vascular structural remodeling is improved by SO_2_ (4). In addition, SO_2_ has been introduced as a regulator of cardiac function (5). Based on a previous study, SO_2_ administration can majorly decrease myocardial infarct. Moreover, SO_2_ can reduce the malondialdehyde (MDA) level in rats, and at the same time, increase the plasma levels of glutathione peroxidase (GPx), superoxide dismutase (SOD), and reduced glutathione (GSH); furthermore, it can improve myocardial expression of the SOD_1_ protein (6). According to recent research, ischemia/reperfusion (I/R) injury remarkably has an influence on the cerebral ischemia cascade also involved in cerebral trauma and stroke. A prolonged and/or severe reduction in the cerebral blood flow (CBF) can cause oxygen deprivation and glucose delivery disorder and accumulation of potentially toxic substances. Considering the high rate of oxidative metabolic activity in the brain, this central organ is susceptible to oxidative stress (7). Oxygen is reintroduced by the returned blood flow, thereby damaging DNAs, plasma membranes, and proteins. On the other hand, cell membrane damage can augment free radical release that caspase cascade activation, redox signaling, and apoptosis of cells are associated with (8). The pyramidal neurons in the CA1 area of the hippocampus are the most sensitive neurons to cerebral ischemia (9). In addition, apoptosis is a major process in CA1 neurons with exposure to transient global ischemia, (10). In cerebral ischemic patients, cognitive functions, including learning and memory, are damaged. Pyramidal neurons of the hippocampus have major involvement in memory and learning processes. In addition, dysfunction of passive avoidance memory following ischemia is associated with CA1 neuronal damage (11). Therefore, considering the underlying mechanisms of brain ischemia and the protective effect that was reported about SO_2_, in the present study for the first time we investigate the effects of SO_2 _administration on memory dysfunction and neuronal damage following transient global cerebral ischemia/reperfusion.

## Materials and Methods

Adult male Wistar rats (n=48) that weighed 260–300 g were obtained from Pasteur Institute, Tehran, Iran. 

The animals were maintained in standard cages with free access to water and food (humidity, 45-50%; temperature, 22–24 ^°^C; 12:12 hr light-dark cycle); the experiments were carried out from 8:00 am to 2:00 pm. We tried to reduce animal suffering and the number of used animals as much as possible. All experiments were in line with the Declaration of Helsinki. 

***Experimental design and protocols***

Four days after ischemia, passive avoidance memory test was carried out in four groups: sham group (n=12), I/R group (n=12), and I/R+SO_2_ groups (NaHSO_3_ and Na_2_SO_3_; 1:3 ratio; 5 and 10 µg/kg, IP, respectively; for 3 days) (6). The same surgical procedures were performed in the sham group; however, no occlusion of common carotid arteries was reported. After behavioral tests, rats were deeply anesthetized by chloral hydrate 400 mg/kg, IP, and then, the brains of half of the animals from each group were removed for biochemical assessments. Brains of other animals after transcardiac perfusion were removed for Nissl and TUNEL staining.

***Transient global cerebral ischemia***

The animals were anesthetized with ketamine (80 mg/kg, IP) and xylazine (10 mg/kg, IP) for inducing transient cerebral ischemia (12). The vagus nerves were separated carefully after exposing and removing the common carotid arteries from the carotid sheath. Yasargil aneurysm clips were used for occluding the common carotid arteries for over 20 min. In a heating system (feedback-regulated), the rectal temperature during surgery was 36.5±0.5 ^°^C. Each rat was left inside the chamber after clipping, and the temperature was 36.5±0.5 ^°^C during occlusion. After releasing and evaluating the carotid arteries for immediate reperfusion, blood flow restoration was confirmed in the carotid arteries via observation. SO_2_ donor (NaHSO_3_ and Na_2_SO_3_; 1:3 ratio; 5 and 10 µg/kg, IP, respectively; single dose per day for 3 days)(6). 

***Passive avoidance task***

Four days after surgery, the passive avoidance memory test was performed using a shuttle box (Borj Sanaat Co, Iran) apparatus**.** It consisted of an illumination chamber made of transparent plastic (30×20×20), as well as a dark chamber with identical dimensions and a dark opaque plastic ceiling and walls. Between the chambers, an opening (8×8 cm) was located, which was closed by an opaque door. The floors were of stainless steel rods (diameter, 2 mm) in both chambers (1 cm apart), and the dark chamber floor could be electrified. The apparatus was in a soundproof room under standard conditions. 

The experiments included the following sessions: adaptation, training, and memory retention. During adaptation, the animals were placed in the illuminated compartment, while having access to the dark compartment. The rats were put in the illuminated compartment following 60 min of adaptation for the purpose of training. The door was closed after the animal’s entrance to the dark compartment, then from the steel-rod floor, they received an electric shock (3 sec, 0.5 mA). 

The final session was memory retention, which was performed 24 hr after the previous session. After placing the animal inside the illuminated compartment, the total time in the dark compartment, as well as step-through latency (STL), was examined. After determining the latency to enter the dark chamber for 3 min, the test was terminated. During the test session, no shock was delivered to the animals (10).

***Biochemical analysis***

After the behavioral assessment, half of the animals were anesthetized and their brains were removed for biochemical analysis, the hippocampal samples (n=6 per group) were homogenized in an ice-cold RIPA buffer containing protease inhibitor and then centrifuged at 3000 g for 20 min at 4 ^°^C. The supernatant was removed and used to evaluate enzymes as follow (13). 

***Measurement of the hippocampal oxidative stress index***

MDA level as a marker of lipid peroxidation was determined as previously described by Ahshin –Majd *et al* (14). For determination of MDA concentration (thiobarbituric acid reactive substances, TBARS), trichloroacetic acid and TBARS reagent were added to the supernatant, mixed, and incubated at boiling water for 90 min. After cooling on ice, samples were centrifuged at 1000 × g for 10 min and the absorbance was read at 532 nm and its final value was obtained from the tetraethoxypropane standard curve, MDA level was measured and expressed as nmol/mg protein.

SOD activity was measured as previously reported. Briefly, the supernatant was incubated with xanthine and xanthine oxidase in potassium phosphate buffer (pH 7.8, 37 ^°^C) for 40 min, and then nitro blue tetrazolium (NBT) was added. Thereafter, blue formazan was monitored spectrophotometrically at 550 nm. The amount of protein that inhibited NBT reduction to 50% maximum was defined as 1 nitrite unit (NU) of SOD activity (15).

GSH is a tripeptide that is capable of preventing damage to important cellular components caused by reactive oxygen species; hippocampus GSH was assayed according to the Griffith method. 5, 50-Dithiobis2-nitrobenzoic acid was used as a chromogen and the absorbance of the reduced chromogen was measured at 412 nm (16).

***Histopathology***

After behavioral test rats were deeply anesthetized by chloral hydrate 400 mg/kg, IP. Then Animals were perfused through the ascending aorta with 200–250 ml saline followed by 200–250 ml of the fixative solution containing 4% paraformaldehyde in 0.1 M phosphate buffer (PH 7.4). Following perfusion, the brains were removed from the skull and were embedded in the same solution for 72 hr. After dehydration in graded concentrations of ethanol, brains were embedded in paraffin blocks for sectioning. In accordance with the Paxinos Atlas, coronal paraffin-embedded sections (7 μm) were cut posterior to the bregma fortune between 3.3 and 4.2 mm for different staining methods (17). Three sections were counted in each brain for histological assay using Image Tool 2 software.

**Figure 1:**
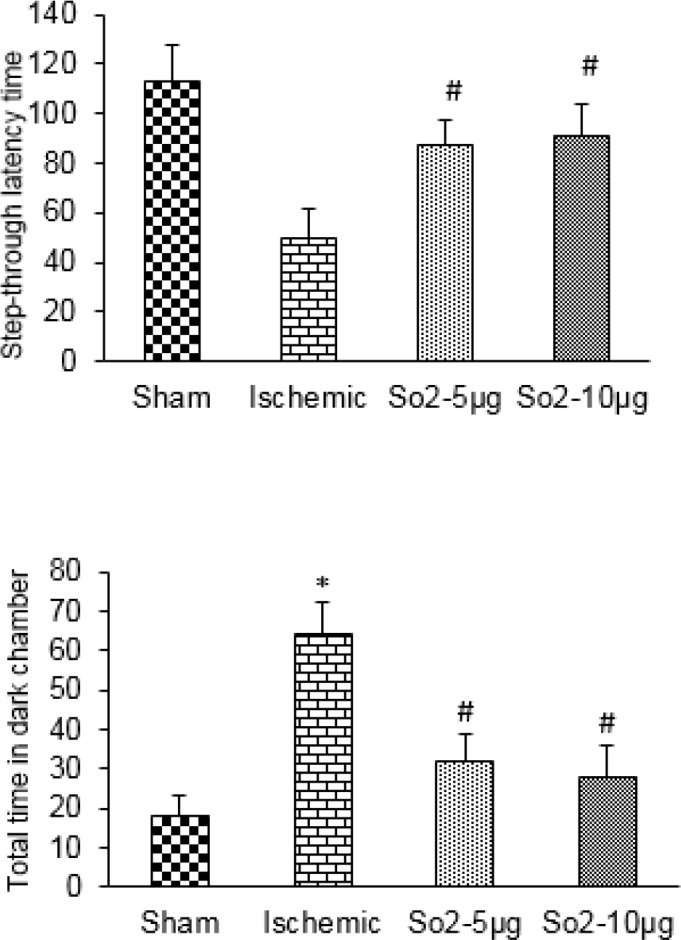
The effects of SO_2_ on step-through latency time and total time spent in the dark chamber during the retention session

* Significantly different compared with the sham group (*P*<0.01)

# Significantly different compared with the ischemia group (*P*<0.05)

**Figure 2:**
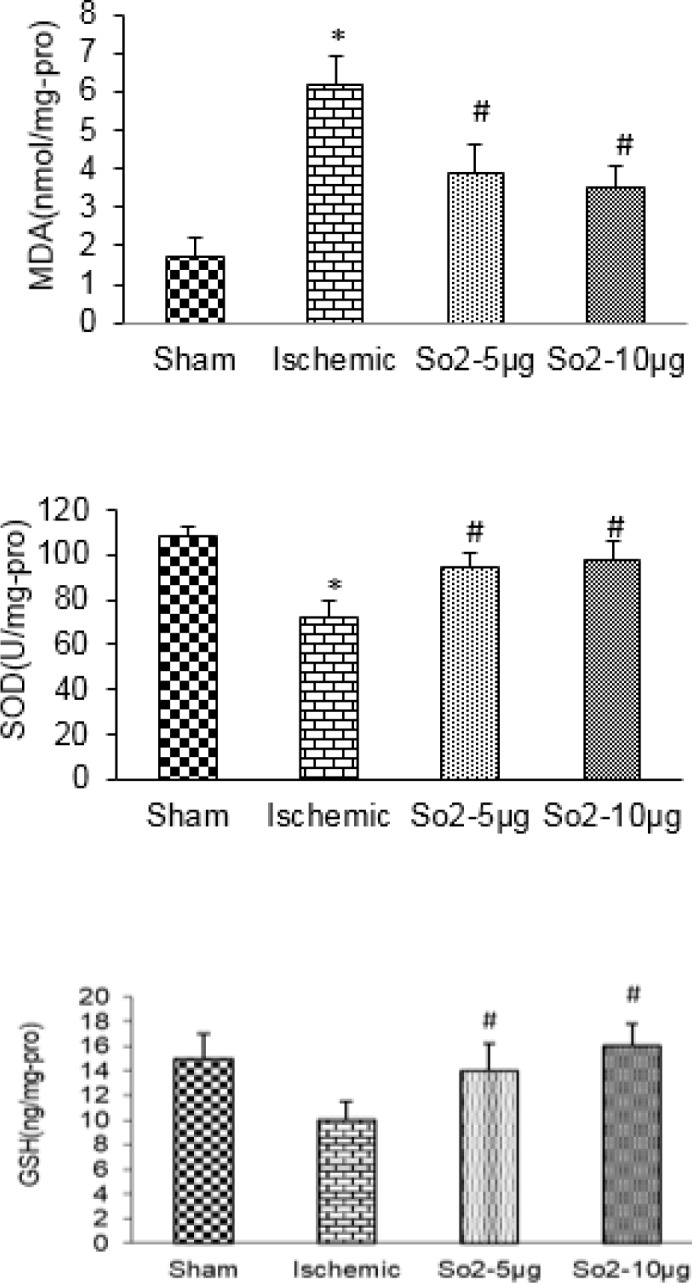
Hippocampus SOD, MDA, and GSH concentration in different groups

**Figure 3:**
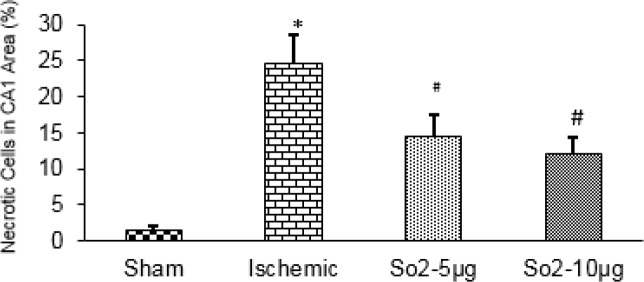
Effects of SO_2_ on ischemia-induced necrotic cell death in the hippocampal CA1 area of rats using Nissl staining. SO_2_ significantly attenuated ischemia-induced necrotic cell death in the CA1 area

* Significantly different compared with the sham group (*P*<0.001)

# Significantly different compared with and the ischemic group (*P*<0.05)

**Figure 4:**
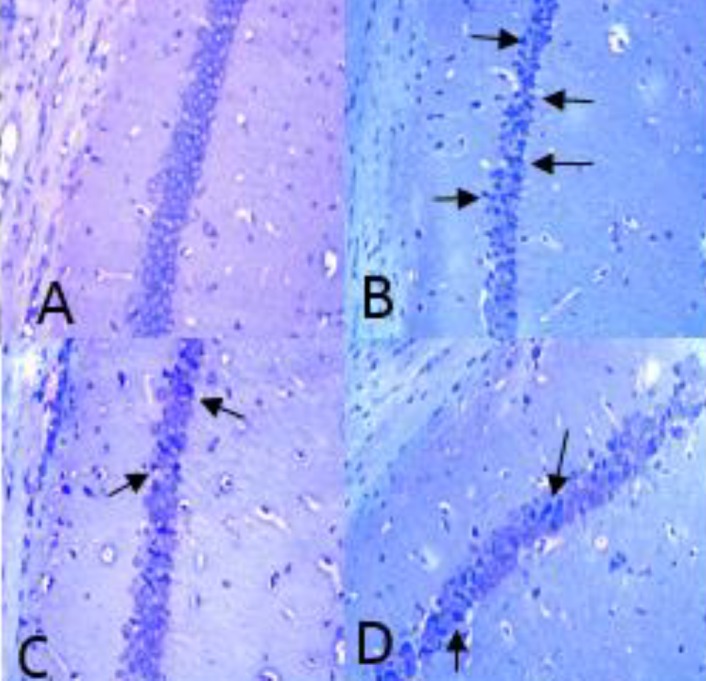
Photomicrographs of Nissl staining in the hippocampus after transient global cerebral ischemia. A: sham group, B: ischemia group, C: SO_2_ treated group 5 µg/kg, D: SO_2_ treated group 10 µg/kg (magnification ×400)

**Figure 5:**
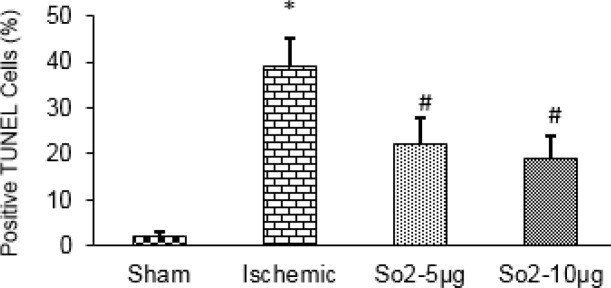
Effects of SO_2_ on ischemia-induced apoptotic cell death in the hippocampal CA1 area of rats. SO_2_ significantly attenuated ischemia-induced apoptotic cell death

* Significantly different compared with the sham group (*P*<0.001)

# Significantly different compared with the ischemic group (*P*<0.01)

**Figure 6:**
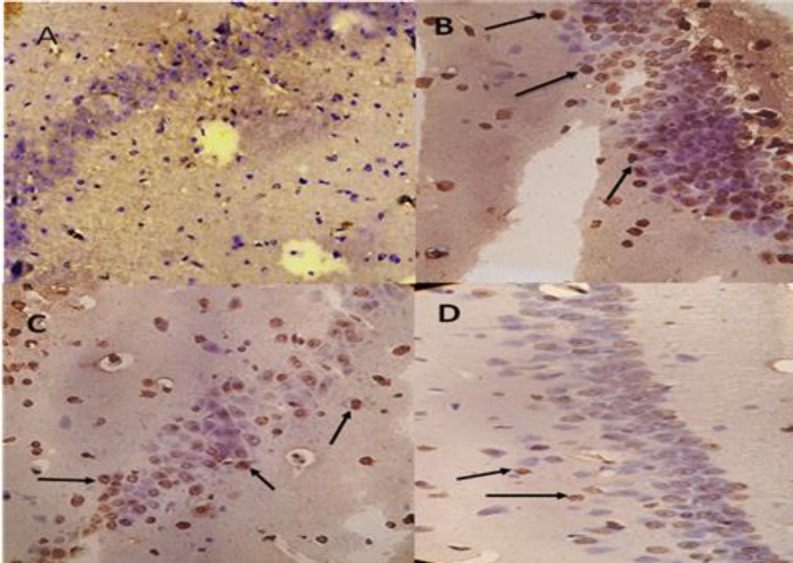
Photomicrographs of TUNEL staining in the hippocampus after transient global cerebral ischemia. A: sham group, B: ischemia group, C: SO_2_ treated group 5 µg/kg, D: SO_2_ treated group 10 µg/kg (magnification ×400)

***Nissl staining***

The basic neuronal structure can be distinguished from necrotic neurons via Nissl staining (17). For staining, the sections (7 μm; 3 sections from each rat) were mounted on gelatin-coated glass slides, followed by air-drying. After staining the slides (cresyl violet 1.0%; Sigma Aldrich), they were dehydrated and coverslipped using Entellan. Afterward, a blinded researcher prepared and measured 3 photomicrographs via light microscopy at ×400 magnification (Olympus AX-70). Then, the ImageTool 2 program was used for analyzing the images. The cells were counted along a 400-µm length (0.160 mm^2^) of the right hippocampal CA1 region (18). 

***TUNEL staining***

Terminal deoxynucleotidyl transferase dUTP nick end labeling (TUNEL) assay was applied for detection of labeling DNA fragmentation in the apoptotic cell death by using an in situ cell death detection kit (Roche, Mannheim, Germany) based on the manufacturer’s protocol. Concisely, after deparaffinization with xylol, the sections (three sections per animal) were rehydrated via descending alcohol series of alcohol and incubated by proteinase K at room temperature. Then, incubation with 3% H_2_O_2_ in methanol was performed for blocking endogenous peroxidase in the dark. Then the TUNEL reaction mixture was added in a humidified atmosphere and 37 ^°^C. After a washing step, Converter-POD was used for 30 min in the dark for visualization, and then the slides were rinsed with PBS, and DAB substrate (0.05% 3, 3-diaminobenzidine) was utilized for 10 min as a chromogen. In addition, hematoxylin was used for counterstaining. Eventually, TUNEL positive cells were quantified using light microscopy (LABOMED USA, magnification 400×). Apoptotic index (the percentage of apoptotic to total cells) cell counts were performed during transect of the 400 µm length of CA1 area of the right hippocampus (18). Three sections were counted in each brain (6 rats in each group) for TUNEL staining.

***Statistical analysis***

Results are expressed as means ± SEM, The statistical significance was determined by using one-way analysis of variance followed by Tukey’s *post-hoc* test. Results were considered to be statistically significant between two groups when *P*<0.05.

## Results

***Effect of sulfur dioxide on passive avoidance memory***

Twenty-four hours after the training session, in the retention session, the latency to cross into the darkened compartment was significantly different between groups. Moreover, a significant reduction in response latency was observed in the ischemic group (50.12±12.1, *P*<0.01) compared to the sham group (112±15); this response latency was significantly increased in the treatment group by SO_2 _compared with the ischemic group (85.23±10.45, *P*<0.05) (Figure 1). Figure 2 shows the effect of SO_2_ injection on the total time spent in the dark chamber during the retention session. The results of this experiment also showed an increase in time spent in the dark chamber in the ischemic group (66.14 ±8.3) compared with the sham group (18.51±5.09 *P*<0.01). This response was significantly decreased in the treatment group by SO_2 _compared with the ischemic group (28.42±7.91 *P*<0.05, Figure 1).

***Effect of SO***_2_ ***on MDA, SOD, and glutathione levels***

To estimate the possible involvement of oxidative stress in hippocampal tissue, MDA level was measured and expressed as nmol/mg protein. Global cerebral ischemia significantly increased hippocampal MDA level in the I/R group in comparison with the sham group (*P*<0.01). Administration of SO_2_ (5, 10 µg/kg) in the I/R group decreased the hippocampal MDA level compared with I/R (*P*<0.05). 

SOD activity was used to determine the level of brain tolerance against the oxidative stress. SOD activity decreased in the hippocampal sample of the I/R group in comparison with the sham group (*P*<0.01) in the I/R group. Administration of SO2 (5, 10 µg/kg) in the I/R group increased the hippocampal SOD level compared with I/R (*P*<0.05). 

Glutathione activity was significantly decreased in the hippocampus of the I/R group (*P*<0.05) in comparison to the sham group. Administration of SO_2 _(5 and 10 µg/kg) in the I/R group increased the hippocampal glutathione level in comparison with I/R (*P*<0.05, Figure 2). 

***SO***_2_ ***attenuated ischemia-induced neuronal necrosis in the hippocampus***

The results of Nissl staining showed that necrotic cells increased in the CA1 area of right hippocampus in the ischemic group (24%±4) rather than in the sham group (2 %± 2, *P*<0.001). SO_2_ treated rats, had decreased necrotic cells versus the ischemia group (13% ± 3 *P*<0. 05, Figure 3).

***Treatment with SO***_2_ ***reduces ischemia-induced apoptotic cell death***

The results of TUNEL staining showed that the percentage of TUNEL-positive cells in the hippocampal CA1 area in the ischemia group (39.8%±5) was significantly increased compared with sham (2%±1.5 *P*<0.001). Also, in ischemic rats treated with SO_2_, the percentage of TUNEL was decreased compared to the ischemia group (19%±4, *P*<0.01, Figure 4).

## Discussion

Based on the findings, SO_2_ donor (5 and 10 µg/kg) could significantly improve memory deficits, while increasing antioxidant enzyme and decreasing neuronal death in hippocampal CA1 pyramidal cells after I/R injury in rats. Moreover, it has been well established that the most important causes of neuronal apoptosis include the caspase pathway, induction of inflammation, free radical formation, disruption in calcium balance, and excitotoxicity (9). It seems that every factor that suppresses this processes can be applied in the treatment of cerebral ischemia. Nesfatin-1 can be one of these factors that multi-functional protective effects have been reported about. SO_2_ is one of the factors which mediates the effects. A possible mechanism for the neuroprotective potential of SO_2_ may be attributed to its ability to block the formation of free radicals. Moreover, at the beginning of the reperfusion after cerebral ischemia, free radical formation is maximized (19). The brain is vulnerable to oxidative stress damage, as neurons contain many polyunsaturated fatty acids, while the endogenous antioxidant enzyme count is low in neuronal tissues (20). 

Increased formation of reactive nitrogen and oxygen species increases neuronal death via damaged DNA, oxidizing proteins, and increased products of lipid peroxidation in the cell membranes (21). The activity of selected antioxidative enzymes (including SOD and GPx) has been shown to increase by SO_2_. Therefore, SO_2 _can reduce DNA damage in cardiac cells and trigger a redox adaptation response, resulting in the antioxidant upregulation and reduced lipid peroxidation (22). The present study also showed that SO_2_ can improve SOD and GSH levels while decreasing MDA.

MDA, which is a cytotoxic product of lipid peroxidation, is recognized as a biomarker of oxidative stress, indicating the production of free radicals and tissue damage (23). Antioxidant enzymes, including SOD and GSH, protect the cells against oxidative damage. They provide a defense mechanism for aerobic organisms to survive. Superoxide anion dismutation into oxygen and hydrogen peroxide is catalyzed by SOD (a metalloenzyme). In addition, GSH protects the cells against damage through reactive oxygen species, including free radicals and peroxides (24).

It was also established that production of the reactive oxygen species (ROS) and disruption of the metabolic balance are the major causes of necrosis (25). It is well-stated that the proapoptotic family proteins including Bax, BaD, and caspase-3 have an important role in triggering of apoptosis (26). On the other hand, the correlation between Bcl-2 expression and resistance to apoptosis is known to result from Bcl-2 properties. For example, Bcl-2 sensitivity to redox changes and its antioxidant functions during calcium stress leads to attenuation of cell death (27).

In addition, the capacity to block the formation of inflammatory cytokines including interleukin-6 (IL-6), interleukin-1 β (IL-1β), and tumor necrosis factor α (TNF-α) increases. Another possible mechanism for the neuroprotective ability of SO_2_ could be enhancement of toxic effects of IL-1β in a synergistic manner due to the increased level of TNF-α. Therefore, brain damage after ischemia is induced by these inflammatory cytokines (28). The increased level of TNF-α is associated with some apoptotic pathways, involved in neuronal death (29). It was shown that SO_2_ has anti-inflammatory effects and decreases above-mentioned cytokine levels in lung injury induced by limb ischemia/reperfusion in rats (30).

## Conclusion

In this study, administration of SO_2_ improves memory impairment induced by I/R. The hippocampus is highly vulnerable to ischemic insults, particularly the pyramidal cells of the CA1 area (17). In addition to neuronal loss in the hippocampus at the time of brain injury, apoptosis process is observed as the continuously delayed neurodegeneration in the following days (31). It appears that the hippocampus has an important role in memory (32). Thus memory deficits that were happening frequently after cerebral ischemia are often related to impaired hippocampal function (33).

Authors from the current investigation conclude that SO_2_ treatment can significantly improve memory impairment following ischemia-induced neuronal damage in the hippocampus of rats via its antioxidant activity and inhibition of necrotic and apoptotic cell deaths.
